# Optimizing anti-TNF therapy in Behçet’s disease: the role of therapeutic drug monitoring

**DOI:** 10.3389/fimmu.2026.1930666

**Published:** 2026-08-03

**Authors:** Marko Barešić, Ljiljana Smiljanić Tomičević, Ana Kozmar, Miroslav Mayer

**Affiliations:** 1 Division of Clinical Immunology and Rheumatology, Department of Internal Medicine, University of Zagreb, School of Medicine, University Hospital Centre Zagreb, Zagreb, Croatia; 2 Department of Laboratory Diagnostics, University Hospital Centre Zagreb, Zagreb, Croatia; 3 Faculty of Pharmacy and Biochemistry, University of Zagreb, Zagreb, Croatia

**Keywords:** adalimumab, anti-drug antibodies, anti-TNF therapy, Behçet’s disease, infliximab, therapeutic drug monitoring, treatment optimization, trough levels

## Abstract

Behçet’s disease (BD) is a complex, relapsing multisystem disorder characterized by autoinflammatory features. Because disease-specific diagnostic biomarkers are lacking and clinical presentation varies according to geographic background and organ involvement, diagnosis may be delayed, particularly in non-endemic regions and in patients with atypical manifestations. Therapeutic management is highly individualized and guided by disease severity and dominant organ involvement. Severe, refractory, or organ-threatening disease often requires aggressive immunosuppressive treatment, including targeted biologic therapy with monoclonal anti-tumor necrosis factor (anti TNF) alpha agents such as infliximab and adalimumab. Therapeutic drug monitoring (TDM), based on serum trough drug concentrations and anti-drug antibodies, is routinely used to optimize anti-TNF therapy in inflammatory bowel disease and is increasingly discussed in other immune-mediated inflammatory diseases treated with monoclonal antibodies. In Behçet’s disease, however, TDM is not yet standard practice, and Behçet-specific therapeutic trough targets for infliximab and adalimumab have not been validated. Nevertheless, integrating TDM with clinical assessment may support more individualized decision-making by helping distinguish insufficient drug exposure, immunogenicity, and pharmacodynamic failure. In this article, we discuss the rationale for TDM in anti-TNF-treated BD, summarize the current direct and indirect evidence, and propose a cautious, clinically guided approach. Behçet’s disease; infliximab; adalimumab; anti-TNF therapy; therapeutic drug monitoring; trough levels; anti-drug antibodies; treatment optimization

## 1 Introduction

Behçet’s disease (BD) is a chronic, relapsing, multisystem inflammatory disorder characterized by recurrent oral aphthae, genital ulcers, skin lesions, ocular inflammation, vascular involvement, neurologic disease, gastrointestinal ulceration, and musculoskeletal symptoms. The clinical course is highly heterogeneous and exhibits overlapping autoimmune and autoinflammatory features. Additionally, the symptoms vary depending on whether patients originate from endemic or non-endemic countries. Some patients experience predominantly mucocutaneous disease, while others develop severe organ-threatening manifestations that may lead to visual loss, intestinal complications, neurologic disability, vascular damage, or substantial impairment of quality of life (1–3). The management of BD is therefore driven by dominant organ involvement, disease severity, relapse pattern, and risk of irreversible damage. Conventional immunosuppressive agents remain important, but anti-tumor necrosis factor (anti-TNF) alpha therapy has become one of the most effective options for patients with refractory or severe disease. Infliximab (IFX) and adalimumab (ADA) are the most commonly used anti-TNF agents in this setting. They have shown benefit in ocular, intestinal, vascular, neurologic, and mucocutaneous BD, and their introduction has improved outcomes for many patients who previously had limited therapeutic options (4, 5).

The 2025 update of the European Alliance of Associations for Rheumatology (EULAR) recommendations for the management of BD, developed by a multidisciplinary task force, provides individualized, evidence-based treatment strategies based on specific organ involvement. The guidelines establish 5 overarching principles and 12 clinical recommendations, maintaining a step-up approach that uses colchicine as a first-line treatment for mucocutaneous and joint manifestations, while advocating for immediate, aggressive intervention with glucocorticoids and immunosuppressives for major organ involvement to prevent irreversible damage. Regarding the use of monoclonal antibodies, the updated guidelines strongly encourage the early application of monoclonal TNF-alpha inhibitors, such as IFX and ADA, for patients suffering from severe, refractory, or life-threatening conditions. Specifically, these monoclonal agents are prioritized for both the rapid induction of remission and long-term maintenance therapy in cases involving sight-threatening posterior uveitis, arterial aneurysms, major venous thrombosis, high-risk gastrointestinal ulcers, and parenchymal nervous system involvement. Additionally, the task force notes that monoclonal TNF-alpha inhibitors may be administered concurrently with conventional synthetic immunosuppressives to optimize disease control, prevent organ relapses, and reduce the risk of immunogenicity (6). There is no mentioning of therapeutic drug monitoring (TDM) strategy in the recommendations.

Despite this therapeutical progress, several clinical challenges remain unresolved. A proportion of patients do not respond adequately to anti-TNF therapy from the beginning. Others achieve remission or partial improvement but later relapse. In daily practice, clinicians often respond to inadequate control empirically by increasing the dose, shortening the dosing interval, adding or modifying concomitant immunosuppression, switching from IFX to ADA or vice versa, or changing to a biologic with the same or different mechanism of action. These decisions are clinically important, particularly in ocular, neurologic, vascular, or intestinal BD, where delayed control of inflammation may result in irreversible damage. Unlike other inflammatory disorders in which anti TNF monoclonal antibodies are used, switching to non TNF biologics is not strongly recommended for refractory Behçet’s syndrome due to insufficient supporting evidence as highlighted in the 2025 update of the EULAR recommendations. Therefore, TDM of anti TNF monoclonal antibodies becomes particularly crucial for ensuring adequate drug exposure and improving clinical outcomes.

This article discusses the potential role of TDM in optimizing anti-TNF therapy in BD. We argue that TDM is not yet ready for universal routine use in all patients with BD, but that selective reactive use may be clinically valuable in specific scenarios, particularly secondary loss of response, suspected immunogenicity, intestinal involvement, and severe organ-threatening disease.

## 2 Therapeutic drug monitoring as a tool for anti-TNF optimization in Behçet’s disease

### 2.1 Principles of therapeutic drug monitoring and mechanisms of anti-TNF failure

TDM is a clinical strategy involving the quantification of serum drug concentrations and the assessment of anti-drug antibodies. This approach is particularly critical for medications with narrow therapeutic windows, such as lithium, cyclosporine, aminoglycoside antibiotics, mycophenolate mofetil and several antiepileptic drugs (7–9). In the management of inflammatory bowel disease (IBD), TDM is primarily utilized to guide dose titration and optimize the efficacy of pharmacotherapy. In the era of monoclonal antibodies, this paradigm is most extensively validated for anti-TNF-alpha agents, principally the monoclonal antibodies IFX and ADA (10).

**Figure 1:**
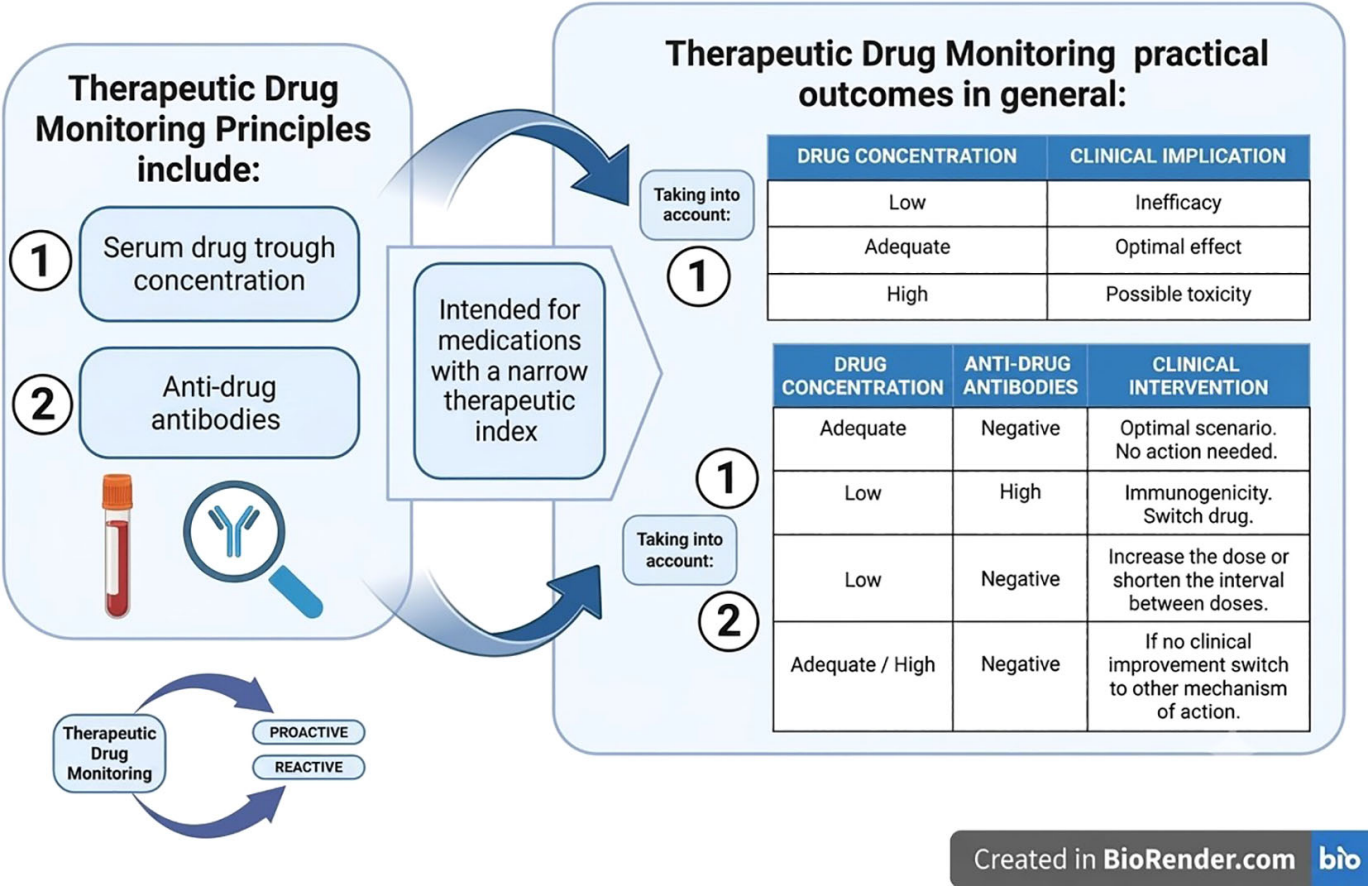
The core principles and clinical utility of therapeutic drug monitoring (TDM).

TDM is clinically useful because treatment failure can arise through different mechanisms. The first mechanism is insufficient drug exposure. A patient may have active disease because serum drug concentrations are too low to control inflammation. Low concentrations may result from increased drug clearance, high inflammatory burden, low albumin, higher body weight, irregular administration, delayed infusions, treatment interruption, or other pharmacokinetic factors. In this situation, increasing the dose or shortening the dosing interval may restore therapeutic exposure. The second mechanism is immunogenicity. Anti-drug antibodies can bind therapeutic monoclonal antibodies, increase clearance, reduce circulating drug concentrations, and contribute to denizloss of efficacy. This issue is particularly relevant for IFX because it is chimeric, but it may also occur with ADA. In immune-mediated inflammatory diseases, concomitant immunosuppressive therapy may reduce immunogenicity in selected settings, although this strategy must be balanced against infection risk, comorbidity, and patient-specific factors. The third mechanism is pharmacodynamic or mechanistic failure. In this situation, drug concentrations are adequate and anti-drug antibodies are absent or not clinically relevant, yet inflammation persists. This suggests that TNF-α blockade is not sufficient to control the patient’s disease. Further dose escalation may be ineffective, and switching to another therapeutic mechanism may be more appropriate. TDM therefore provides a decision-making framework. Rather than viewing all anti-TNF failure as a single clinical problem, clinicians can classify failure as likely pharmacokinetic, immunogenic, or pharmacodynamic (11). This classification is especially valuable in Behçet’s disease because empirical decisions may expose patients to unnecessary dose escalation, delayed switching, avoidable adverse effects, or prolonged uncontrolled inflammation. The core principles of TDM are shown in **Figure 1**.

### 2.2 Reactive and proactive TDM: current clinical relevance

The use of TDM is generally categorized into reactive and proactive strategy. Reactive TDM involves measuring drug concentrations and anti-drug antibodies in response to a clinical event, such as a disease flare, primary non-response, or secondary loss of response. It is well-established and recommended in most clinical guidelines for managing IBD. For anti-TNF therapies, reactive TDM is considered more cost-effective than empiric dose optimization (10, 12). Proactive TDM involves regular, scheduled monitoring of drug levels and antidrug antibodies regardless of clinical symptoms, with the goal of maintaining concentrations within a target therapeutic range to prevent future flares. Evidence regarding proactive TDM is evolving, and guideline recommendations remain divergent. TDM strategy is not yet incorporated in the treatment protocol for BD.

**Figure 2:**
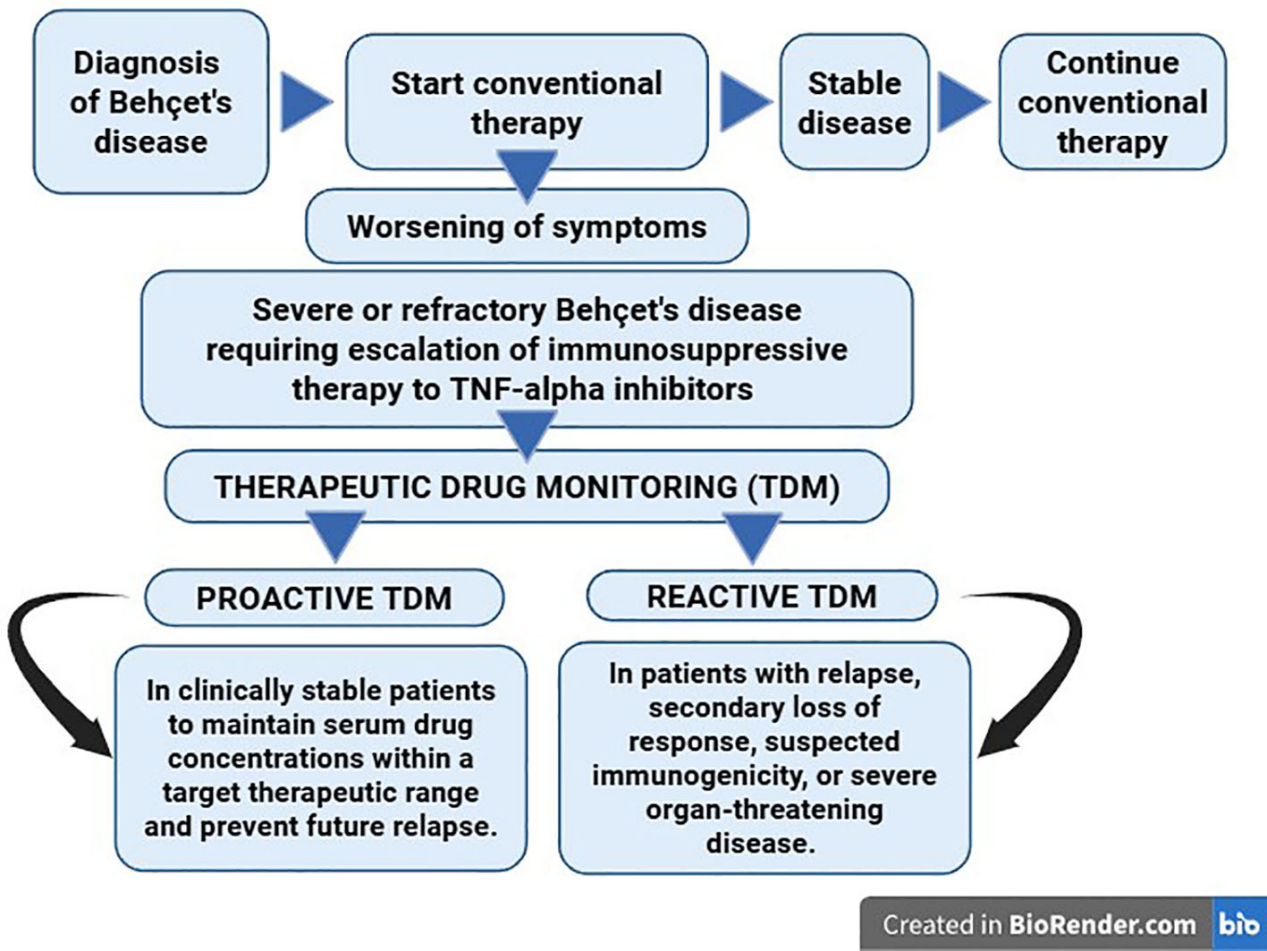
Decision-making framework based on TDM.

There are no validated Behçet-specific IFX or ADA trough targets, and it is unknown whether therapeutic thresholds should differ between ocular, intestinal, neurologic, vascular, and mucocutaneous disease. Therefore, proactive therapeutic drug monitoring cannot currently be recommended as a routine strategy for all patients with Behçet’s disease. Reactive therapeutic drug monitoring is more defensible. It does not require the same level of certainty about ideal target levels because the main question is often practical: is treatment failure associated with low drug exposure, anti-drug antibodies, or active disease despite adequate exposure? Even without Behçet-specific targets, this information may help guide management in selected patients. Decision-making framework based on TDM is shown in **Figure 2**.

### 2.3 Anti-TNF therapy in Behçet’s disease

TNF alpha is an important mediator of inflammation in Behçet’s disease. Anti-TNF therapy targets this pathway and has become a major therapeutic option in severe or refractory disease (4). IFX is a chimeric monoclonal antibody administered intravenously or subcutaneously, while ADA is a fully human monoclonal antibody administered subcutaneously. Both agents neutralize TNF-α and reduce inflammatory activity. The strongest clinical experience with anti-TNF therapy in BD comes from ocular involvement, especially posterior uveitis and panuveitis. In these patients, rapid suppression of inflammation is crucial because repeated attacks can lead to irreversible visual impairment. Anti-TNF therapy has also been used successfully in intestinal BD, neurologic involvement, vascular disease, and refractory mucocutaneous or articular manifestations. Multicenter cohorts and reviews describe high response rates in severe or refractory BD, although the evidence is limited by heterogeneity, small sample sizes, retrospective designs, and lack of standardized outcome measures (4, 5).

Phenotype is also central to treatment optimization. BD is not a single-organ disorder, and the clinical urgency differs between phenotypes. Ocular, neurologic, vascular, and intestinal disease may require rapid and sustained suppression of inflammation. Mucocutaneous and articular disease can also be disabling, but the risk of irreversible organ damage is usually different. Emerging comparative data suggest that anti-TNF choice and durability may vary according to phenotype, with ADA potentially more durable in mucocutaneous and ocular disease, while IFX may offer faster control in vascular and neurologic disease. These findings require further confirmation, but they illustrate a key principle: anti-TNF optimization in BD must begin with organ phenotype, not drug levels alone (13).

### 2.4 Lessons from inflammatory bowel disease and Behcet-specific gaps

The strongest evidence for TDM comes from IBD. In Crohn’s disease and ulcerative colitis, low IFX or ADA trough concentrations and anti-drug antibodies are associated with loss of response in a proportion of patients. Reactive therapeutic drug monitoring is used to guide dose escalation, interval shortening, switching within anti-TNF class, or switching out of class (12, 14). This evidence is particularly relevant to intestinal BD. In inflammatory bowel disease, up to 30% of patients may show no initial benefit from anti-TNF therapy, and a substantial proportion lose response during follow-up, often because of low drug concentrations or anti-drug antibodies rather than pure mechanistic failure (12). In intestinal BD, anti-TNF failure has also been reported, and baseline endoscopic severity together with early inflammatory markers may identify patients at higher risk of failure (8).

However, caution is necessary. BD has unique systemic features, including vascular inflammation, neurologic involvement, recurrent mucocutaneous disease, and ocular relapses. A trough concentration sufficient for luminal intestinal healing may not be sufficient for posterior uveitis, neurologic disease, or vascular inflammation. Conversely, concentrations derived from inflammatory bowel disease may overestimate what is needed for some mucocutaneous phenotypes. Therefore, inflammatory bowel disease provides a useful conceptual framework, but not a complete Behçet-specific protocol. Direct evidence for TDM in BD remains sparse. The literature supports the efficacy of IFX and ADA in severe and refractory BD, but does not establish validated serum trough targets or anti-drug antibody thresholds (4, 5). The limited Behçet-specific evidence available suggests that immunogenicity is not a major limitation of anti-TNF therapy. In the largest controlled study, anti-infliximab antibodies were detected in only 6% of patients and were associated with lower trough drug concentrations, disease relapses, and infusion reactions, whereas most patients without antibodies maintained long-term treatment response (15). More recent real-world data demonstrated high drug-retention rates for both infliximab and adalimumab, with concomitant azathioprine use in over 60% of patients, which may have contributed to reduced immunogenicity and sustained treatment persistence. Adalimumab was associated with longer relapse-free survival, whereas infliximab provided more rapid disease control in severe vascular and neurological phenotypes (13). Nevertheless, these findings originate from a limited number of observational studies, and prospective studies are still needed to define Behçet-specific therapeutic targets and clarify the role of immunogenicity in treatment optimization. The current evidence therefore supports a balanced position. Anti-TNF optimization is clinically important in BD, and therapeutic drug monitoring may be useful in selected situations. However, Behçet-specific therapeutic ranges, assay standards, and prospective outcome data are still lacking.

### 2.5 Proposed clinical use of TDM in BD

At present, TDM in BD should be selective, reactive, and phenotype-aware. It may be considered in patients with secondary loss of response after initial improvement, relapse during maintenance therapy, suspected immunogenicity, infusion or injection reactions, persistent inflammation despite apparently adequate treatment, or severe organ-threatening disease where rapid optimization is essential.

A practical interpretation can be adapted from IBD, while recognizing that it is not validated specifically for BD.

Low drug concentration without anti-drug antibodies suggests insufficient exposure. Management may include checking adherence, reviewing dosing delays, assessing inflammatory burden, increasing the dose, or shortening the dosing interval.

Low or undetectable drug concentration with anti-drug antibodies suggests immunogenic failure. Management may include switching to another anti-TNF agent, considering concomitant immunosuppression when appropriate, or switching to another biologic mechanism depending on disease phenotype and urgency. Adequate drug concentration with active disease suggests pharmacodynamic failure. In this situation, repeated escalation may have limited benefit, and switching to another mechanism should be considered.

Adequate drug concentration with clinical remission supports continuation of the current regimen. In selected patients with sustained remission, cautious dose reduction or interval extension may be considered, although evidence for de-escalation in BD is limited.

## 3 Discussion

TDM may offer a more individualized approach to the treatment strategy of BD patients. By measuring serum drug concentrations and anti-drug antibodies, therapeutic drug monitoring can help determine whether treatment failure is related to inadequate drug exposure, immunogenicity, or persistent inflammation despite adequate drug levels. This distinction has practical therapeutic consequences. Low drug levels without anti-drug antibodies may support dose escalation or interval shortening. Low drug levels with anti-drug antibodies may suggest immunogenic failure and support switching therapy. Adequate drug levels with active disease may indicate pharmacodynamic failure and support switching to another therapeutic mechanism.

TDM is not yet a standard component of BD management, but it has a potential of becoming one in the near future in the context of precise and individualized medicine. Its greatest current value is likely in reactive use, especially in patients with relapse, secondary loss of response, suspected immunogenicity, or severe organ-threatening disease. In these situations, therapeutic drug monitoring may reduce empirical decision-making and help clinicians choose between dose escalation, interval shortening, switching within class, or switching to another mechanism.

The most evidence-based position seems to be cautious extrapolation from IBD and related immune-mediated inflammatory diseases, particularly for intestinal BD. However, extrapolation should not be confused with validation. BD has distinct pathophysiology and phenotype-specific therapeutic goals. Therefore, drug concentrations considered adequate in inflammatory bowel disease may not necessarily be adequate for ocular, neurologic, or vascular BD.

TDM should therefore be used as part of individualized care. It should not replace clinical assessment, organ-specific monitoring, or expert judgment. Instead, it should be interpreted alongside disease phenotype, severity, inflammatory markers, imaging, endoscopy, ophthalmologic evaluation, treatment history, and patient-specific factors.

## 4 Conclusion

Anti-TNF therapy is effective and clinically important in severe and refractory BD, but treatment failure and relapse remain major challenges. TDM offers a rational framework for distinguishing insufficient drug exposure, immunogenicity, and pharmacodynamic failure. However, Behçet-specific evidence remains limited, and validated trough targets for infliximab and adalimumab have not yet been established. Reactive TDM seems to be a potentially promising option in patients with loss of response by addressing a fundamental clinical dilemma whether the loss of response is driven by subtherapeutic drug exposure, immunogenicity, or active disease persisting despite adequate drug levels. At present, TDM should be considered selectively, particularly in patients with secondary loss of response, relapse during maintenance therapy, suspected immunogenicity, intestinal involvement, or severe organ-threatening disease requiring rapid optimization. Future prospective studies should define phenotype-specific targets, clarify the role of anti-drug antibodies, and determine whether TDM-guided treatment improves remission, treatment persistence, relapse prevention, and long-term organ outcomes. Until then, TDM should be viewed not as a rigid protocol, but as a useful adjunct to individualized, phenotype-driven clinical decision-making in BD.
